# Antibiotic Susceptibility Profiles of Lactic Acid Bacteria from the Human Vagina and Genetic Basis of Acquired Resistances

**DOI:** 10.3390/ijms21072594

**Published:** 2020-04-08

**Authors:** Auttawit Sirichoat, Ana Belén Flórez, Lucía Vázquez, Pranom Buppasiri, Marutpong Panya, Viraphong Lulitanond, Baltasar Mayo

**Affiliations:** 1 Departamento de Microbiología y Bioquímica, Instituto de Productos Lácteos de Asturias (IPLA-CSIC), Paseo Río Linares, s/n, 33300 Villaviciosa, Asturias, Spain; auttawit_s@kkumail.com (A.S.); abflorez@ipla.csic.es (A.B.F.); lucia.vazquez@ipla.csic.es (L.V.); 2 Department of Microbiology and Research and Diagnostic Center for Emerging Infectious Diseases (RCEID), Faculty of Medicine, Khon Kaen University, Khon Kaen 40002, Thailand; viraphng@kku.ac.th; 3 Instituto de Investigación Sanitaria del Principado de Asturias (ISPA), Avenida de Roma s/n, 33011 Oviedo, Spain; 4 Department of Obstetrics and Gynecology, Faculty of Medicine, Khon Kaen University, Khon Kaen 40002, Thailand; bprano@kku.ac.th; 5 College of Medicine and Public Health, Ubon Ratchathani University, Ubon Ratchathani 34190, Thailand; marutpong.p@ubu.ac.th

**Keywords:** antibiotic resistance, tetracycline resistance, genome analysis, vaginal microbiota, lactic acid bacteria, *Lactobacillus*, *Bifidobacterium bifidum*

## Abstract

Lactic acid bacteria can act as reservoirs of antibiotic resistance genes that can be ultimately transferred to pathogens. The present work reports on the minimum inhibitory concentration (MIC) of 16 antibiotics to 25 LAB isolates of five *Lactobacillus* and one *Bifidobacterium* species from the human vagina. Acquired resistances were detected to kanamycin, streptomycin, chloramphenicol, gentamicin, and ampicillin. A PCR analysis of lactobacilli failed to identify genetic determinants involved in any of these resistances. Surprisingly, a *tet*(W) gene was detected by PCR in two *Bifidobacterium bifidum* strains, although they proved to be tetracycline-susceptible. In agreement with the PCR results, no acquired genes were identified in the genome of any of the *Lactobacillus* spp. strains sequenced. A genome analysis of *B. bifidum* VA07-1AN showed an insertion of two guanines in the middle of *tet*(W) interrupting the open reading frame. By growing the strain in the presence of tetracycline, stable tetracycline-resistant variants were obtained. An amino acid substitution in the ribosomal protein S12 (K43R) was further identified as the most likely cause of VA07-1AN being streptomycin resistance. The results of this work expand our knowledge of the resistance profiles of vaginal LAB and provide evidence for the genetic basis of some acquired resistances.

## 1. Introduction

The extensive use of antibiotics has led to the emergence, evolution and spread of antibiotic resistance (AR) in pathogenic and non-pathogenic bacteria associated with humans, animals and the environment [1]. The rapid spread of AR among pathogens coupled with the lack of new antibiotics is a major global health concern [2]. The selective pressure imposed by the presence of antibiotics in the environment has driven the spread of AR by horizontal gene transfer (HGT) events [3]. The antibiotics used to treat infections in humans are commonly the same as those used in veterinary medicine, which has resulted in the rapid dissemination of AR genes among bacteria associated with the food chain [4].

Lactic acid bacteria (LAB) form part of the indigenous microbiota of food, but also that of human and animal mucosa. Many LAB strains are used as starter and adjunct cultures to control food fermentations, while others are employed as probiotics for the prevention and treatment of intestinal diseases [5]. Although phylogenetically unrelated, bifidobacteria, which are found in the same ecological niches as LAB and are used for the same applications, have—for practical reasons—traditionally been included among the true LAB [5]. The European Food Safety Authority (EFSA) has awarded most LAB species ‘Qualified Presumption of Safety (QPS)’ status [6], and they are ‘Generally Recognized as Safe (GRAS status)’ by the U.S. Food and Drug Administration (FDA) [7]. The only criterion that LAB strains have to fulfill to meet QPS requirements is the absence of AR genes against antimicrobials of clinical and veterinary importance [8]. Although LAB are non-pathogenic, they can act as reservoirs of AR genes which might eventually be transferred via HGT to pathogenic bacteria during food manufacture or after consumption [9].

Lactobacilli are among the most dominant populations in the vaginal ecosystem together with other LAB species (e.g., bifidobacteria) [10,11]. These bacteria exist in finely tuned mutualistic relationships with each other and with the host, providing the first line of defense against colonization and infection by pathogens [12]. Selected LAB strains could potentially be used as probiotics for preventing or treating vaginal infections [13]. In order to prevent the spread of AR in the vaginal ecosystem, any strain used as a probiotic must be free of acquired AR genes with transference capability. It is important to note that some LAB strains, including some from the human vagina, have already been reported to carry transferable AR genes [9,14,15,16,17].

The present work characterizes members of the vaginal microbiota of healthy Thai women as part of the search for probiotic candidate strains to be used in the vaginal ecosystem. Dominant LAB from vaginal exudates, all of which produced large amounts of lactic acid, were recovered in culture and identified by molecular methods. The antibiotic resistance/susceptibility profiles of 25 isolates belonging to different LAB species are here reported, along with information on the genetic basis of the acquired resistances encountered.

## 2. Results

### 2.1. Isolation, Identification and Typing of Vaginal LAB

Twenty-five vaginal LAB isolates with clear acidification halos on MRS agar supplemented with 0.5% CaCO_3_ were recovered. All were Gram-positive rods, catalase negative, and γ-hemolytic. They were subsequently identified at the species level by 16S rRNA gene sequencing and sequence comparison. Seven isolates were identified as belonging to *Lactobacillus crispatus*, six to *Lactobacillus salivarius*, four to *Lactobacillus jensenii*, four to *Lactobacillus paracasei,* two to *Lactobacillus reuteri,* and two to *Bifidobacterium bifidum* (Table 1). A fingerprinting analysis by combining the results of RAPD-PCR and rep-PCR techniques detected 21 different strains among the 25 isolates, distributed as follows: *L. crispatus* (six strains), *L. salivarius* (five), *L. jensenii* (three), *L. paracasei* (three)*, L. reuteri* (two)*,* and *B. bifidum* (two strains) (Supplementary Figure S1).

### 2.2. Antibiotic Susceptibility

Table 1 shows the MIC values of the 16 tested antibiotics for the 25 vaginal LAB isolates. All isolates were phenotypically susceptible to tetracycline, erythromycin, clindamycin, penicillin, quinupristin-dalfopristin, linezolid and rifampicin. The distribution of neomycin MICs covered more than nine 2-fold dilutions, ranging from ≤0.5 to 256 µg mL^−1^. Similarly, a wide distribution of MICs was observed for streptomycin (≤0.5 to >256 µg mL^−1^), ciprofloxacin (≤0.25 to 64 µg mL^−1^), and chloramphenicol (1 to 8 µg mL^−1^). The most common resistance phenotypes observed were those to trimethoprim (MIC 16 to ≥64 µg mL^−1^) and vancomycin (MIC ≥ 128 µg mL^−1^). Nine isolates were resistant to kanamycin (MIC values 32 to >1024 µg mL^−1^). The two *B. bifidum* strains were susceptible to all tested antibiotics except for streptomycin (MIC >256 µg mL^−1^). Moderate resistance to chloramphenicol was seen in three isolates and for ampicillin in one isolate. Interestingly, *L. salivarius* VA40-10 was highly resistant to all four aminoglycosides tested (gentamicin, kanamycin, streptomycin, and neomycin).

### 2.3. Detection of AR Genes by PCR

**Figure 1:**
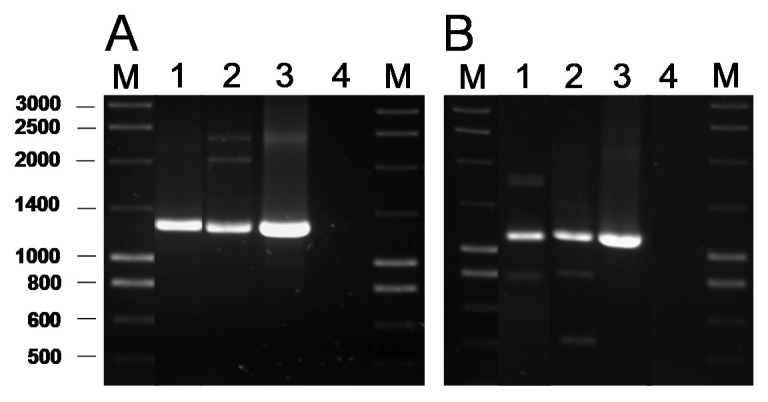
PCR amplification of tetracycline resistance genes using the universal primers Tet1 and Tet2 targeting a segment of 1,300 bp of the genes encoding RPP (**A**) and 1,200 bp of the *tet*(W) gene with the specific primer pair tetWF-Tet2 (**B**). Key of samples: Lane 1, DNA from *B. bifidum* VA07-1AN; lane 2, *B. bifidum* VA07-2AN; lane 3, *Leuconostoc mesenteroides* subsp. *mesenteroides* LbE16 (positive control) [9]; line 4, blank (no template DNA). M, molecular weight marker.

The presence of genes coding for the commonest AR genes spread among LAB was investigated by PCR. No genes involved in resistance to chloramphenicol (*cat*), β-lactams (*bla*), aminoglycosides [*aac(6′)-aph(2″)* and *aad*(E)], macrolides [*erm*(A), *erm*(B), *erm*(C), *erm*(F), *mef*(A)], tetracycline [*tet*(M), *tet*(O), *tet*(S), *tet*(K), *tet*(L)], clindamycin (*lsaA*) or vancomycin (*vanA*) were ever detected in any of the isolates (data now shown). In contrast, a PCR analysis for genes encoding ribosomal protection proteins (RPP) causing tetracycline resistance using the degenerate primer pairs DI-DII and Tet1-Tet2 (Supplementary Table S1) produced a positive amplification when DNA from two tetracycline-susceptible *B. bifidum* isolates was used as a template (Figure 1A). Amplification with gene-specific primers gave a positive result only for the *tet*(W) gene (Figure 1B). Amplicon sequencing and sequence comparison further proved the presence in these isolates of a *tet*(W) gene highly homologous to those present in many Gram-positive and Gram-negative bacteria.

### 2.4. Genome Analysis for AR Genes

Based on the phenotype and genotype results (Table 1; Supplementary Figure S1), six strains were subjected to genome sequencing: *L. crispatus* VA50-4AN (resistant to kanamycin, ampicillin, and trimethoprim), *L. jensenii* VA04-2AN (resistant to trimethoprim), *L. salivarius* VA40-10 (resistant to gentamicin, kanamycin, streptomycin, neomycin, and vancomycin), *L. paracasei* VA02-1AN (resistant to chloramphenicol and vancomycin), *L. reuteri* VA24-5 (resistant to vancomycin and trimethoprim), and *B. bifidum* VA07-1AN (resistant to streptomycin). Supplementary Table S2 shows the general features of their genomes. Their size was, in all cases, around 2.2 Mbp but the number of contigs obtained after assembly ranged from 17 to 300. Supplementary Table S3 summarizes some of the key genetic features of the genomes of the sequenced strains. Genes coding for penicillin binding proteins (PBP) were found in all the genomes, although with different numbers and types for the distinct species. Mutations in PBPs encoding-genes known to confer AR were not identified. One gene coding for a D-alanine-D-alanine ligase (Ddl) was detected in each of the strains. In several LAB species, the presence of phenylalanine at the enzyme active site in Ddl is correlated with intrinsic resistance to vancomycin [18]. In addition, in each of the strains, 9-32 genes were classified by the RAST server as belonging to the category “Virulence, Disease, and Defence”, subcategory “Resistance to Antibiotic and Toxic Compounds”. The majority of these genes encoded components dedicated to homeostasis or resistance to heavy metals, such as copper, mercury, and the cobalt-zinc-cadmium triad. Genes encoding elongation factors, efflux pumps, DNA gyrases, and topoisomerases were also included by RAST in this subcategory.

**Figure 2:**
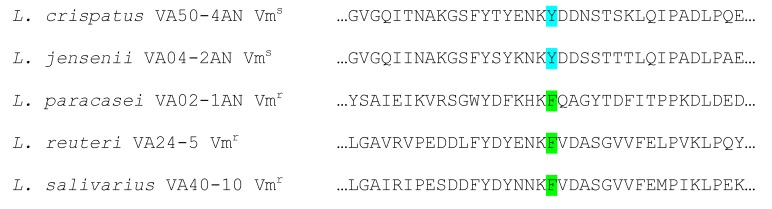
Alignment of amino acid sequences around the active site of D-Ala-D-Ala ligases of the five *Lactobacillus* spp. strains sequenced. Strains with phenylalanine (F) at the enzyme active site (green) show a vancomycin-resistant phenotype, while those having a tyrosine (Y) (pale blue) display a vancomycin-susceptible phenotype.

By comparing the genome sequences against the databases CARD, ResFinder, and ARG-ANNOT, no genes known to be involved in AR in *L. jensenii* VA04-2AN (resistant to trimethoprim), *L. paracasei* VA02-1AN (resistant to chloramphenicol and vancomycin), and *L. reuteri* VA24-5 (resistant to trimethoprim and vancomycin) were detected. The only positive correlation between phenotype and genotype was the presence of a conserved phenylalanine (F) residue in the active site of the Ddl ligase, corresponding to amino acid 261 of the *Leuconostoc mesenteroides* enzyme [18], in the deduced sequence of all vancomycin-resistant (Vm^r^) strains, while the susceptible (Vm^s^) strains were characterized by the presence of a tyrosine (Y) residue at this position (Figure 2).

Genome analysis of *L. crispatus* VA50-4AN, *L. salivarius* VA40-10, and *B. bifidum* VA07-1AN identified no genes known to be involved in aminoglycoside resistance. Therefore, mutations in key genes, such as those coding for the ribosomal S12 protein and others acting on the 16S rRNA molecule, were therefore sought by comparing the DNA and deduced protein sequences from our strains with those in databases. No amino acid differences were observed in the sequences of the ribosomal protein S12 for *L. crispatus* VA50-4AN and *L. salivarius* VA40-10 from those belonging to susceptible strains of the same species. Further, alignment of the deduced amino acid sequences for the 16S rRNA guanine(527)-N(7)-methyltranferase (RsmG) proteins of the sequenced strains, showed heterogeneity at several positions between themselves and with respect to sequences in databases. In particular, the RsmG sequence of *L. crispatus* VA50-4AN showed one amino acid change at position 38 (N→H), while that of *B. bifidum* VA07-1AN showed three amino acid changes at positions 105 (E→A), 150 (G→D), and 206 (R→G), and that of *L. salivarius* VA40-10 showed six exclusive amino acid changes at positions 12 (G→E), 67 (D→N), 186 (N→D), 199 (I→V), 208 (Q→K), and 209 (V→I). However, by comparing RsmG sequences from resistant and susceptible strains, none of the changes considered could be associated with streptomycin resistance.

**Figure 3:**
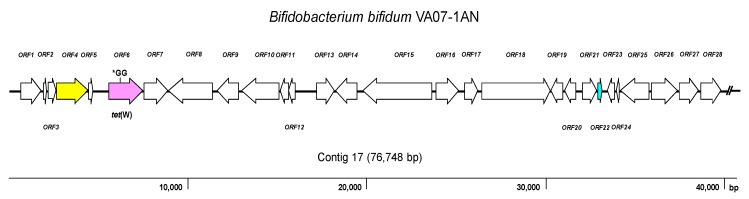
Diagram showing the genetic organization of ORFs in the contig harboring the *tet*(W) gene of *Bifidobacterium bifidum* VA07-1AN. Color key: purple, *tet*(W) gene (the position of the GG insertion disrupting the ORF is indicated); yellow, conjugation-associated gene; pale blue, gene encoding a transcription regulator; white, genes involved in other processes. The broken line symbol indicates the contig extends beyond this point.

As expected, the genome analysis confirmed the presence of *tet*(W) in *B. bifidum* VA07-1AN; this gene was also unequivocally identified by searches in the three AR databases used. The *tet*(W) gene in *B. bifidum* VA07-1AN was located in a contig of 76,748 bp. Figure 3 shows the genetic organization of the 40-kbp left extreme of the contig that included the *tet*(W) gene. The *tet*(W) sequence of VA07-1AN (1922 bp) was almost identical to that described for *Bifidobacterium longum* LTBL16 (CP034089.1). Similar *tet*(W) sequences have also been found in the chromosome of strains belonging to other species such as *B. bifidum* L22 (NG_048301.1), *Lachnospiraceae* bacterium KGMB03038 (CP041667.1), and *Ruminococcus* sp. JE7A12 (CP039381.1); and in plasmids, such as pTZC1 from *Cutibacterium acnes* TP-CU389 (LC473083.1). Compared to *tet*(W) in *B. longum* LTBL16, the *tet*(W) in *B. bifidum* VA07-1AN contained an insertion of two extra guanine residues (GG) after nucleotide 731 in the ORF resulting in a frameshift, which produced only a short peptide—289 amino acids long compared to 639 residues for the functional Tet(W). This likely explains the susceptibility of VA07-1AN to tetracycline. The *tet*(W) gene was flanked by ORFs coding for proteins showing the greatest homology to others from *B. longum* in the upstream region, and proteins typical of *B. bifidum* in the downstream region (Supplementary Table S4).

**Figure 4:**
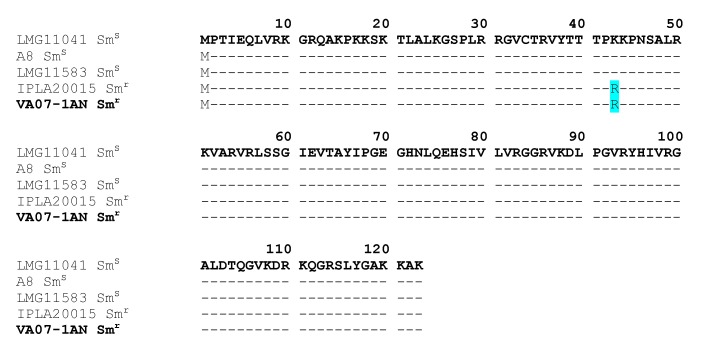
Alignment of the deduced amino acid sequence of S12 ribosomal proteins encoded by the *rpsL* gene from streptomycin resistant (Sm^r^) and susceptible (Sm^s^) *Bifidobacterium bifidum* strains. The amino acid replacement K→R at position 43 in the resistant strains is highlighted in pale blue. In bold, the strain of this study (VA07-1AN).

The CARD database further identified in the genome of *B. bifidum* VA07-1AN a single nucleotide polymorphism (SNP) point mutation in the *rpsL* gene (encoding the ribosomal S12 protein), a variation causing an amino acid substitution (K→R) at position 43 of the protein (Figure 4). This amino acid change has been associated with streptomycin resistance in many species [19].

### 2.5. Restoration of the Tetracycline Resistance Phenotype in B. Bifidum VA07-1AN

**Figure 5:**
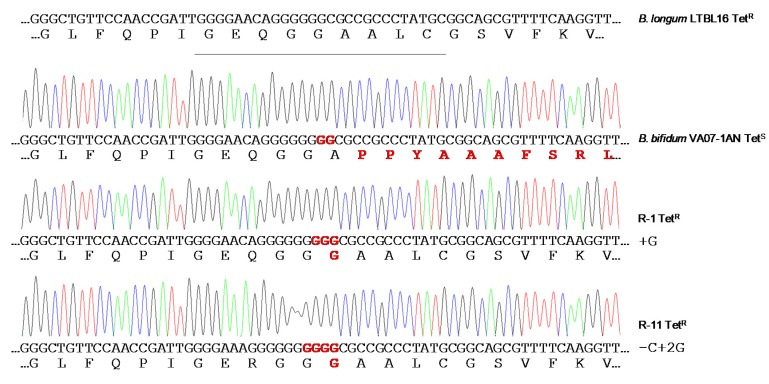
Chromatograms of amplicons of the *tet*(W) gene from the original tetracycline-susceptible strain *B. bifidum* VA07-1AN and two representative tetracycline-resistant revertants (R-1 and R-11). Nucleotide sequences and the corresponding deduced amino acid sequences are displayed below each of the chromatograms. DNA and protein differences with canonical sequences of the *tet*(W) gene from the tetracycline-resistant *Bifidobacterium longum* LTBL16 strain (on top of the figure) are highlighted in red.

When the susceptibility of *B. bifidum* VA07-1AN to tetracycline was assayed using the MICE test, colonies growing within the inhibition halo were noted. Identification and typing showed them to be tetracycline-resistant variants of VA07-1AN. After plating on antibiotic-containing and antibiotic-free plates, about 0.6% of the colonies from an overnight culture were found to be tetracycline-resistant. Amplification and sequencing of *tet*(W) genes from 13 tetracycline-resistant variants showed the addition of one guanine nucleotide in most revertants to the guanine stretch where the two Gs disrupting the ORF had been inserted (as in R-1; Figure 5). Other mutations consisting in both nucleotide insertions and deletions in the vicinity of the stretch of Gs were occasionally seen (as in R-11; Figure 5). In either case, there was a net gain of one nucleotide, which, together with the two Gs that disrupted the *tet*(W) ORF, created a new codon that opened the reading frame of Tet(W) producing a functional protein that provided tetracycline resistance. The MIC of tetracycline in the tetracycline-resistant variants ranged from 48 to 96 μg mL^−1^. In contrast, growing the antibiotic-resistant variants in the absence of tetracycline for about 80-100 generations showed no tetracycline-susceptible revertants, demonstrating a high stability of the mutations that restored the resistant phenotype.

## 3. Discussion

LAB contribute to the maintenance of vaginal health via the production of substances (mainly organic acids) that acidify the environment and inhibit the development of pathogens [13]. However, there is an increasing concern that LAB may act as reservoirs of AR determinants, from which they could ultimately be transferred to pathogens [3,20]. Indeed, the existence of lactobacilli and bifidobacteria strains resistant to several antibiotics, by either acquiring mutations or exogenous genes, has been repeatedly reported [21,22,23,24,25]. Therefore, during the selection of probiotics, the susceptibility of lactobacilli and bifidobacteria to antibiotics has to be assessed and the absence in the selected strains of transferable AR genes should be assured [26].

Studies reporting lactobacilli and bifidobacteria to be generally susceptible to tetracycline, erythromycin, chloramphenicol, penicillin, ampicillin, clindamycin, quinupristin-dalfopristin, linezolid and rifampicin have been published over the last 15 years [24,27,28]. In agreement, the phenotypic analysis of the present isolates showed them to be susceptible to these antibiotics, with the exception of ampicillin and chloramphenicol—to which one and three isolates, respectively, were associated with MIC values higher than EFSA’s cut-offs [8]. In contrast, nine isolates showed resistance to one or more aminoglycosides (gentamicin, kanamycin, streptomycin, and neomycin). Resistance to aminoglycosides may occur based on several mechanisms, which include (i) enzymatic modification and inactivation of the antibiotics mediated by aminoglycoside acetyltransferases, nucleotidyltransferases, or phosphotransferases, (ii) increased efflux, (iii) decreased permeability, and (iv) modifications of the 30S ribosomal subunit interfering with the binding of this class of antibiotics [29]. However, most of the MIC values recorded in this study were just one dilution higher than the corresponding cut-off. These small MIC differences might be explained by the normal variation associated with the microdilution assay [30]. Accordingly, none of the aminoglycoside resistance genes searched for by PCR, including the widespread *aac*(6′)-*aph*(2″) and *aad*(E) genes [31], were found in any of the isolates. The genome analysis further discarded the presence of acquired resistances in the sequenced strains, comprising genes and well-characterized mutations involved in aminoglycoside resistance. Given the lack of cytochrome-mediated drug transport, aminoglycoside resistance has been claimed an intrinsic feature of LAB and other anaerobic bacteria [32]. However, large differences in the MIC values for aminoglycosides even in strains from the same species have been reported in the literature [24,27,28]. The cooperation of other non-specific mechanisms, such as increased membrane impermeability, enhanced activity of unspecific efflux pumps and multi-drug transporters, or presence of defective cell wall autolytic systems, may further account for differences in MICs between different species and strains [33].

Resistance to the aminoglycoside streptomycin has largely been associated with mutations in chromosomal genes, for example, in *rpsL* that codes for the ribosomal protein S12 [34], or in *rsmG* that codes for the 16S rRNA guanine(527)-N(7)-methyltranferase (RsmG) [35]. Comparison of the deduced proteins from streptomycin-susceptible and -resistant strains of the different lactobacilli species analyzed revealed random differences between the RsmG sequences. However, none of them could be consistently associated with streptomycin resistance. In contrast, a mutation in *rpsL* causing an amino acid change at position 43 (K→R) was observed in *B. bifidum* VA07-1AN. The same amino acid replacement has been reported in other streptomycin-resistant strains of bifidobacteria [36] and many other species [19], suggesting this to be the most likely explanation for the high resistance to streptomycin shown by VA07-1AN.

Strong resistance to vancomycin is an intrinsic feature in certain *Lactobacillus* phylogroups and other LAB species such as *Leuconostoc* spp. [37] caused by an amino acid replacement in the active site of the DdlA ligase (F261Y), as it has been experimentally demonstrated for *Leuconostoc mesenteroides* [18] and *L. reuteri* [38].

Although cut-offs for trimethoprim and ciprofloxacin in LAB and bifidobacteria have yet to be defined, strains of most of the present species were associated with quite high MICs. In fact, the resistance of most *Lactobacillus* species to these antibiotics has been repeatedly reported [24,28]. Folate auxotrophy in lactobacilli is generally accepted as the intrinsic cause of resistance to trimethoprim [39]. Similarly, reduced affinity of DNA gyrase (GyrA) and topoisomerase IV (ParC) variants for ciprofloxacin and other fluoroquinolones seen in some LAB species has been determined responsible for their insensitivity to this class of antibiotics [40]. Further, the presence of active multidrug efflux systems could contribute to an increase in the MIC for ciprofloxacin in some strains [21]. Since no genes coding for β-lactamases have ever been detected in LAB, non-specific mechanisms, as already discussed for the aminoglycosides, might contribute to the increased MIC of ampicillin in *L. crispatus* VA50-4AN, as has been reported for *L. reuteri* [41].

The vaginal lactobacilli in the present study were very susceptible to tetracycline—even though many LAB strains are resistant to it [23,31]. Unexpectedly, PCR analysis detected the presence of *tet*(W) in the two tetracycline-susceptible *B. bifidum* strains. This gene has been found to be disseminated among gut-dwelling bacteria of different species from humans and animals [42]. The genome analysis of *B. bifidum* VA07-1AN showed the gene to contain an insertion of two Gs bases at its center, shifting the ORF and rendering a shorter non-functional peptide. The presence of silent tetracycline resistance genes in bifidobacteria has been reported elsewhere [43,44]. The reactivation of a silent tetracycline resistance phenotype has also been reported for *Bifidobacterium animalis* subsp. *lactis* Bb12 [44]. Silent AR genes could therefore represent a hazard, even more so when they can be easily reactivated and the restored gene remains stable afterwards. Therefore, the use of strains harboring such genes in food and feed systems should be avoided.

## 4. Materials and Methods

### 4.1. Sample Selection and Collection

This study was approved by the Khon Kaen Ethics Committee in Human Research (HE581191). Vaginal exudates were obtained from healthy women attending the gynecological outpatient clinic of the Srinagarind Hospital, Faculty of Medicine, Khon Kaen University, Thailand. To be included, subjects had to be 20–45 years old, not be pregnant, have regular menstruation, have no underlying diseases, have a healthy vagina (showing no clinical symptoms upon examination by a gynecologist), and not be taking antibiotics. The lateral, anterior, and posterior vaginal walls were swabbed with a sterile cotton-tipped applicator (performed by a gynecologist). Samples were then placed in a sterile container prepared with Amies transport medium (Oxoid, Basingstoke, UK) and kept on ice until delivery to the laboratory.

### 4.2. Isolation of Lactic Acid Bacteria (LAB)

The collected samples were serially diluted with de Man, Rogosa and Sharpe (MRS) broth (Oxoid) and plated on MRS agar plates containing 0.5% (*w*/*v*) CaCO_3_ (BDH, Poole, UK). These were then incubated under aerobic and anaerobic conditions at 37 °C for 48 h. For anaerobic incubations, liquid or solid MRS medium was supplemented with 0.3 g L^−1^ L-cysteine-HCl (Merck, Darmstadt, Germany) giving rise to MRS-Cys. After incubation, individual colonies surrounded by clear acidification halos were randomly selected, purified on MRS agar with or without cysteine (depending on their aerobic or anaerobic isolation), and examined according to colony morphology, Gram staining, catalase reaction, and hemolytic activity using sheep blood agar plates (Oxoid). Catalase negative, γ-hemolytic, Gram-positive rods were deemed to be LAB and were stored at −80 °C in MRS broth supplemented with 15% (*v*/*v*) glycerol (Merck).

### 4.3. Identification of Bacteria by 16S rRNA Gene Sequencing

Total genomic DNA from isolates was purified from overnight cultures using the GenElute™ Bacterial Genomic DNA kit (Sigma-Aldrich, St. Louis, MO., USA) following the supplier’s recommendations. Purified genomic DNA was used as a template to amplify a large part of the 16S rRNA gene using the universal primers pair 27F (5′-AGAGTTTGATCCTGGCTCAG-3′) and 1492R (5′-GGTTACCTTGTTACGACTT-3′). The PCR conditions were as follows: one cycle at 95 °C for 5 min, 35 cycles at 94 °C for 30 s, 55 °C for 45 s, and 72 °C for 2 min, and a final extension cycle at 72 °C for 10 min. Amplicons were purified using GenElute™ PCR Clean-Up columns (Sigma-Aldrich) and sequenced. Sequences were compared against those in the NCBI database using the Blast program (https://blast.ncbi.nlm.gov/Blast.cgi), and against those in the Ribosomal Database Project database (https://rdp.cme.msu.edu/). Sequences sharing a percentage identity ≥97% to those in databases were considered to belong to the same species.

### 4.4. Molecular PCR Fingerprinting 

Isolates were genetically typed by random amplification of polymorphic DNA-PCR (RAPD-PCR) with primer M13 (5′-GAGGGTGGCGGTTCT-3′) as reported by Rossetti and Giraffa [45] and primer OPA18 (5′-AGGTGACCGT-3′) as reported by Mättö et al. [46], and by repetitive element palindromic PCR (rep-PCR) using primer BoxA2R (5′-ACGTGGTTTGAAGAGATTTTCG-3′), as reported by Koeuth et al. [47]. The PCR reaction mixtures contained 2 µL of each purified genomic DNA (≈100 ng), 12.5 µL of Taq DNA Polymerase 2x Master Mix RED (Ampliqon, Odense, Denmark), 5 µL of primer (10 µM), and molecular biology grade water (Sigma-Aldrich) in a total volume of 25 µL. The PCR conditions were: one cycle at 95 °C for 7 min, 40 cycles at 95 °C for 30 s, annealing for 30 s at 42 °C for primer M13, 40 °C for primer BoxA2R, or 32 °C for primer OPA18, 72 °C for 4 min, and a final extension cycle at 72 °C for 10 min. PCR profiles were visualized in 2.5% agarose gels after 90 min of electrophoresis at 75 V and photographed under UV light. The banding patterns were clustered using the unweighted pair group method with arithmetic mean (UPGMA), and their pattern similarity expressed via the simple matching (SM) coefficient using GeneTools software v.4.03 (SynGene, Cambridge, UK). The results of the triplicate typing analyses of the isolates by the RAPD-PCR and rep-PCR techniques were 94% reproducible. Consequently, profiles with ≥94% similarity were considered to be the same strain.

### 4.5. Antibiotic Susceptibility Testing

The minimum inhibitory concentration (MIC) of 16 antibiotics (Table 1) was determined by broth microdilution according to ISO IDF Standard 10932 [48] using VetMIC plates for LAB (SVA, Uppsala, Sweden). Briefly, individual colonies of the isolates were grown on LAB susceptibility test medium (LSM) [49] agar plates or LSM supplemented with cysteine, and then suspended in 2 mL of a 0.9% sterile saline solution to obtain a density corresponding to McFarland standard 1 (≈3 × 10^8^ cfu mL^−1^). The suspension was further diluted 1000-fold in LSM, and then 100 µL of this suspension were placed in each well of the VetMIC plates. These were then incubated at 37 °C under anaerobic conditions and examined after 48 h incubation. MICs were defined as the concentration at which no visible growth was observed. For some antibiotics, the concentration range of the plates was insufficient to determine the actual MIC for some isolates; these were determined using broth microdilution of the required antibiotics according to the guidelines of the Clinical Laboratory Standards Institute [50], or using the MICE system (Oxoid) following the manufacturer’s recommendations. Resistant strains were distinguished from susceptible strains based on the cut-off values reported by the European Food Safety Authority [8].

### 4.6. PCR Detection and Identification of AR Genes

Tetracycline resistance genes coding for ribosomal protection proteins (RPPs) were searched for in all isolates by using the universal primers DI-DII [51] and Tet1-Tet2 [52] ( Supplementary Table S1). When a positive result was obtained, additional PCR assays were performed with gene-specific primer pairs for *tet*(M), *tet*(S), *tet*(O) [26], and *tet*(W) [53]. Isolates were also tested for the presence of genes coding for the tetracycline efflux pumps *tet*(K) and *tet*(L) [54]. AR genes providing resistance to erythromycin (*erm*(A), *erm*(B), *erm*(C) [55], *erm*(F) [56]), and *mef*(A) [57], chloramphenicol (*cat*) [58], β-lactam antibiotics (*bla*) [58], and aminoglycoside (*aac(6′)-aph(2″)* [59] and *aad*(E) [60]) were also searched for by standard PCR. The PCR conditions used in each case were those described by the corresponding authors (Supplementary Table 1). In addition, a specific primer pair was designed to amplify genes involved vancomycin (*vanA*) resistance (Supplementary Table S1). As positive controls, DNA from appropriate strains of our collection carrying well characterized AR genes was used as a template in the different amplification reactions together with that of the strains of this study. When detected, the amplified PCR products were purified and sequenced. Similarities with sequences held in databases were sought using the Blast program as above.

### 4.7. Genome Sequencing, Annotation, and Analysis

Independent genomic libraries of 0.5 kbp were constructed from the total DNA of six antibiotic resistant strains, and paired-end sequenced at Eurofins Genomics (Konstanz, Germany) using an Illumina HiSeq 1000 System sequencer. Quality-filtered reads were assembled in contigs using Spades software v.3.6.2. (http://cab.spbu.ru/software/spades/). Genomes were annotated using the RAST annotation system (http://rast.nmpdr.org/) and the NCBI Prokaryotic Genome Annotation Pipeline (http://www.ncbi.nlm.nih.gov/genome/annotation_prok/). DNA and deduced protein sequences of interest were examined individually for homology against non-redundant DNA and protein databases using the Blast program. The homology of DNA and proteins was further investigated by searching the antibiotic resistance databases CARD (http://arpcard.mcmaster.ca/), ResFinder (https://cge.cbs.dtu.dk/services/ResFinder/) and ARG-ANNOT (https://www.mediterranee-infection.com/acces-ressources/base-de-donnees/arg-annot-2/).

### 4.8. Stability of the Disrupted tet(W) Gene of B. Bifidum

To check the stability of the tetracycline-susceptible phenotype in *B. bifidum* VA07-1AN, the strain was grown overnight in MRS-Cys broth at 37 °C in anaerobiosis. Dilutions of the cultures were plated on MRS-Cys agar with and without tetracycline. Under the same culture conditions, tetracycline-resistant revertants with a repaired *tet*(W) gene were transferred in liquid MRS-Cys without antibiotic for 5 days (80–100 generations). Dilutions of the cultures were the plated in MRS-Cys agar and the colonies replicated in MRS-Cys plates with and without tetracycline.

### 4.9. GenBank Accession Numbers

Genome sequences were deposited in GenBank under BioProject PRJNA604908 with biosample accession numbers SAMN14087162, SAMN14087187, SAMN14087200, SAMN14087229, SAMN14087235, and SAMN14087237.

## 5. Conclusions

The present results reveal some vaginal LAB showing acquired resistance to several antibiotics. PCR amplification and genome analysis allowed us to uncover the genetic basis of most resistances. Some of these acquired resistances appear to be due to non-specific mechanisms (increased activity of efflux pumps, higher cell-wall impermeability) or to mutations in housekeeping genes, although an acquired AR gene, *tet*(W)—silent but still recoverable—was identified in one strain. This stresses the importance of checking both the phenotype and genotype of LAB when selecting candidates for probiotics.

## Figures and Tables

**Table 1:** Minimum inhibitory concentration (MIC) values of 16 antibiotics to the vaginal LAB species and strains of this study.

Species	Strain	Antibiotic ^a^ (MIC as µg mL^−1^)															
		GEN	KAN	STR	NEO	TET	ERY	CLI	CHL	AMP	PEN	VAN	QDA	LIN	TMP	CIP	RIF
*L. crispatus*	VA20-32AN ^b^	2	16	2	16	2	0.06	0.25	4	1	0.5	0.5	1	4	>64	16	1
	VA27-7	4	32	64	8	1	1	2	8	2	2	1	1	4	32	64	4
	VA27-9	1	16	2	2	2	0.03	0.5	4	2	0.5	0.5	1	4	64	32	2
	VA28-12	1	16	2	2	2	0.06	0.5	4	2	0.5	0.5	2	4	64	32	2
	VA32-17	2	64	2	8	4	0.03	0.5	2	1	1	0.5	1	2	>64	64	8
	VA32-17AN	4	128	32	4	2	0.25	0.5	4	1	0.5	1	1	2	16	32	4
	**VA50-4AN ^c^**	≤0.5	32	1	2	4	0.12	0.12	4	4	1	0.5	1	4	>64	32	4
*L. jensenii*	VA04-1AN	≤0.5	4	2	1	0.25	≤0.016	0.12	4	0.25	0.12	1	0.5	1	>64	8	0.25
	**VA04-2AN**	≤0.5	4	4	1	0.5	0.03	0.12	2	0.5	1	1	0.5	2	>64	8	0.25
	VA15-2AN	≤0.5	≤2	1	≤0.5	1	≤0.016	≤0.03	2	0.06	0.06	0.5	0.5	0.5	>64	8	0.25
	VA16-11	≤0.5	8	1	2	4	0.06	0.25	4	0.06	≤0.03	2	0.5	2	>64	16	0.5
**Breakpoint (µg mL^−1^) ^d^**		**16**	**16**	**16**	**-**	**4**	**1**	**4**	**4**	**2**	**-**	**2**	**-**	**-**	**-**	**-**	**-**
*L. salivarius*	VA09-4	8	64	16	4	2	0.25	0.25	2	1	0.25	128	0.25	0.5	≤0.12	1	2
	VA16-20	≤0.5	4	2	0.5	1	0.06	0.06	2	0.5	0.12	>128	0.5	0.5	0.25	0.5	0.5
	VA37-13	≤0.5	4	≤0.5	≤0.5	0.5	0.06	0.06	2	0.25	0.12	>128	0.5	0.5	0.25	≤0.25	0.5
	**VA40-10**	128	>1024	>256	256	2	1	1	4	1	0.25	>128	1	1	1	4	0.5
	VA40-12AN	4	128	32	4	2	0.25	0.25	4	0.5	0.25	>128	1	0.5	0.25	1	1
	VA40-14AN	4	128	32	4	2	0.25	0.5	4	0.5	0.25	>128	1	0.5	≤0.12	1	1
**Breakpoint (µg mL^−1^)**		**16**	**64**	**64**	**-**	**8**	**1**	**4**	**4**	**4**	**-**	**n.r.**	**-**	**-**	**-**	**-**	**-**
*L. paracasei*	**VA02-1AN**	≤0.5	16	8	1	2	0.12	0.06	8	1	0.25	>128	1	4	0.5	4	0.5
	VA24-4	1	16	8	4	4	0.12	0.06	4	0.5	0.25	>128	1	2	0.25	4	0.5
	VA26-3	≤0.5	16	8	2	2	0.12	0.06	4	1	0.25	>128	1	2	1	2	0.5
	VA27-8	1	32	16	8	2	0.06	0.06	8	0.5	0.25	>128	1	4	0.25	4	0.5
**Breakpoint (µg mL^−1^)**		**32**	**64**	**64**	**-**	**4**	**1**	**4**	**4**	**4**	**-**	**n.r.**	**-**	**-**	**-**	**-**	**-**
*L. reuteri*	VA15-3	≤0.5	4	2	≤0.5	8	0.12	≤0.03	4	1	2	>128	1	2	>64	32	0.25
	**VA24-5**	≤0.5	16	4	≤0.5	16	0.06	≤0.03	4	2	8	>128	0.5	4	>64	32	0.25
**Breakpoint (µg mL^−1^)**		**8**	**64**	**64**	**-**	**32**	**1**	**4**	**4**	**2**	**-**	**n.r.**	**-**	**-**	**-**	**-**	**-**
*B. bifidum*	**VA07-1AN**	8	64	>256	16	1	≤0.016	0.06	1	≤0.03	≤0.03	0.5	0.5	0.5	16	8	2
	VA07-2AN	32	64	>256	32	1	≤0.016	≤0.03	1	≤0.03	≤0.03	1	0.5	0.5	16	8	1
**Breakpoint (µg mL^−1^)**		**64**	**-**	**128**	**-**	**8**	**1**	**1**	**4**	**2**	**-**	**2**	**-**	**-**	**-**	**-**	**-**

^a^ GEN, gentamicin; KAN, kanamycin; STR, streptomycin; NEO, neomycin; TET, tetracycline; ERY, erythromycin; CLI, clindamycin; CHL, chloramphenicol; AMP, ampicillin; PEN, penicillin; VAN, vancomycin; QDA, quinupristin-dalfopristin; LIN, linezolid; TMP, trimethoprim; CIP, ciprofloxacin; RIF, rifampicin. ^b^ Strains coded with AN were recovered after growth in anaerobiosis. ^c^ Strains in bold were subjected to whole genome sequencing and analysis. ^d^ Gray-shaded boxes show MIC of antibiotics higher than the corresponding cut-off values, considering those of the European Food Safety Authority [8]. n.r., naturally resistant; -, breakpoint not established.

## Supplementary Materials

Supplementary materials can be found at https://www.mdpi.com/1422-0067/21/7/2594/s1.
