# Supplementary material for: Antibiotic Susceptibility Profiles of Lactic Acid Bacteria from the Human Vagina and Genetic Basis of Acquired Resistances
Source: Int J Mol Sci. 2020 Apr 8;21(7):2594. doi: 10.3390/ijms21072594 (PMC7178285; doi:10.3390/ijms21072594)
Supplement: Supplementary file 1 [file ijms-21-02594-s001.pdf]

Supplementary Table 1.- Primers and PCR conditions for amplifying selected antibiotic resistance genes used in this study.

Resistance gene	Primers	Sequence (5' – 3')	*Tm* (°C)	Amplicon size (bp)	Reference
RPP[a]	DI<br>DII	GAYACNCCNGGNCAYRTNGAYTT<br>GCCCARWANGGRTTNGGNGGNACYTC	50	1,083	(Clermont et al., 1997)
RPP	Tet-1<br>Tet-2	GCTCACGTTGACGCAGGAA<br>AGGATTTGGCGGGACTTCTA	50	1,300	(Barbosa et al., 1999)
tet(M)	DI<br>tetM_R	GAYCANCCNGGNCAYRTNGAYTT<br>CACCGAGCAGGGATTTCTCCAC	55	1,513	(Gevers et al., 2003)
tet(O)	tetO_F<br>tetO_R	AATGAAGATTCCGACAATTT<br>CTCATGCGTTGTAGTATTCCA	45	781	(Gevers et al., 2003)
tet(S)	tetS_F<br>tetS_R	ATCAAGATATTAAGGAC<br>TTCTCTATGTGGTAATC	45	573	(Gevers et al., 2003)
tet(W)	tetWF<br>Tet-2	AAGCGGCAGTCACTTCCTTCC<br>AGGATTTGGCGGSACTTCTA	50	1,200	(Scott et al., 2000)
tet(K)	tetK_F<br>tetK_R	TTATGGTGGTTGTAGCTAGAAA<br>AAAGGGTTAGAAACTCTTGAAA	45	348	(Gevers et al., 2003)
tet(L)	tetL_F<br>tetL_R	GTMGTTGCGCGCTATATTCC<br>GTGAAMGRWAGCCCACCTAA	45	696	(Gevers et al., 2003)
erm(A)	ermA_F<br>ermA_R	TCTAAAAAGCATGTAAAAGAA<br>CTTCGATAGTTTATTAATATTAGT	48	645	(Rizzotti et al., 2005)
erm(B)	ermB_F<br>ermB_R	GAAAAGGTACTCAACCAAATA<br>AGTAACGGTACTTAAATTGTTTAC	50	639	(Rizzotti et al., 2005)
erm(C)	ermC_F<br>ermC_R	TCAAAACATAATATAGATAAA<br>GCTAATATTGTTTAAATCGTCAAT	43	642	(Rizzotti et al., 2005)
erm(F)	ermF_F<br>ermF_R	CGGGTCAGCACTTTACTATTG<br>GGACCTACCTCATAGACAAG	50	466	(Roberts et al., 1999)
mef(A)	mefA_F<br>mefA_R	ACCGATTCTATCAGCAAAG<br>GGACCTGCCATTGGTGTG	43	940	(Luna et al., 2000)
cat	cat_F<br>cat_R	ATGACTTTTAATATTATTRAWTT<br>TCATYTACMYTATSAATTATAT	49	648	(Hummel et al., 2007a)
bla	bla_F<br>bla_R	CATARTTCCGATAATASMGCC<br>CGTSTTTAACTAAGTATSGY	51	297	(Hummel et al., 2007b)
aac(6')-aph(2")	aac_F<br>aac_R	CCAAGAGCAATAAGGGCATA<br>CACTATCATAACCACTACCG	60	220	(Rojo-Bezares et al., 2006)
aad(E)	aadE_F<br>aadE_R	GCAGAACAGGATGAACGTATTCG<br>ATCAGTCGGAACTATGTCCC	55	369	(Klare et al., 2007)
vanA	vanA_F<br>vanA_R	TCACCCCTTTAACGCTAATAC<br>GTTTGGGGGTTGCTCAGACC	50	1,006	This study

[a]RPP, genes encoding ribosomal protecting proteins.

Reference

- Barbosa, T.M., Scott, K.P., and Flint, H.J. (1999). Evidence for recent intergeneric transfer of a new tetracycline resistance gene, *tet*(W), isolated from *Butyrivibrio fibrisolvens*, and the occurrence of *tet*(O) in ruminal bacteria. *Environ Microbiol* 1(1), 53-64. doi: 10.1046/j.1462-2920.1999.00004.x.
- Clermont, D., Chesneau, O., De Cespédès, G., and Horaud, T. (1997). New tetracycline resistance determinants coding for ribosomal protection in streptococci and nucleotide sequence of *tet*(T) isolated from *Streptococcus pyogenes* A498. *Antimicrob Agents Chemother* 41(1), 112-116.
- Gevers, D., Danielsen, M., Huys, G., and Swings, J. (2003). Molecular characterization of *tet*(M) genes in *Lactobacillus* isolates from different types of fermented dry sausage. *Appl Environ Microbiol* 69(2), 1270-1275. doi: 10.1128/aem.69.2.1270-1275.2003.
- Hummel, A., Holzapfel, W.H., and Franz, C.M. (2007a). Characterisation and transfer of antibiotic resistance genes from enterococci isolated from food. *Syst Appl Microbiol* 30(1), 1-7. doi: 10.1016/j.syapm.2006.02.004.
- Hummel, A.S., Hertel, C., Holzapfel, W.H., and Franz, C.M. (2007b). Antibiotic resistances of starter and probiotic strains of lactic acid bacteria. *Appl Environ Microbiol* 73(3), 730-739. doi: 10.1128/AEM.02105-06.
- Klare, I., Konstabel, C., Werner, G., Huys, G., Vankerckhoven, V., Kahlmeter, G., Hildebrandt, B., Müller-Bertling, S., Witte, W., and Goossens, H. (2007). Antimicrobial susceptibilities of *Lactobacillus*, *Pediococcus* and *Lactococcus* human isolates and cultures intended for probiotic or nutritional use. *J Antimicrob Chemother* 59(5), 900-912. doi: 10.1093/jac/dkm035.
- Luna, V.A., Cousin, S., Jr., Whittington, W.L., and Roberts, M.C. (2000). Identification of the conjugative *mef* gene in clinical *Acinetobacter junii* and *Neisseria gonorrhoeae* isolates. *Antimicrob Agents Chemother* 44(9), 2503-2506. doi: 10.1128/aac.44.9.2503-2506.2000.
- Rizzotti, L., Simeoni, D., Cocconcelli, P., Gazzola, S., Dellaglio, F., and Torriani, S. (2005). Contribution of enterococci to the spread of antibiotic resistance in the production chain of swine meat commodities. *J Food Prot* 68(5), 955-965. doi: 10.4315/0362-028x-68.5.955.
- Roberts, M.C., Chung, W.O., Roe, D., Xia, M., Marquez, C., Borthagaray, G., Whittington, W.L., and Holmes, K.K. (1999). Erythromycin-resistant *Neisseria gonorrhoeae* and oral commensal *Neisseria* spp. carry known rRNA methylase genes. *Antimicrob Agents Chemother* 43(6), 1367-1372.
- Rojo-Bezares, B., Sáenz, Y., Poeta, P., Zarazaga, M., Ruiz-Larrea, F., and Torres, C. (2006). Assessment of antibiotic susceptibility within lactic acid bacteria strains isolated from wine. *Int J Food Microbiol* 111(3), 234-240. doi: 10.1016/j.ijfoodmicro.2006.06.007.
- Scott, K.P., Melville, C.M., Barbosa, T.M., and Flint, H.J. (2000). Occurrence of the new tetracycline resistance gene *tet*(W) in bacteria from the human gut. *Antimicrob Agents Chemother* 44(3), 775-777. doi: 10.1128/aac.44.3.775-777.2000.

Supplementary Table 2.- General data of the genome sequencing projects of vaginal LAB strains resistant to antibiotics isolated from human.

Species	Strain	Antibiotic resistance	Assembly condition	Nº contigs	Total bases	Contig Max	N50	N90
L. crispatus	VA50-4AN	Kanamycin, ampicillin, and trimethoprim resistant	Spades (t145)	300	2,215,244	131,661	25,648	6,043
L. salivarius	VA40-10	Gentamicin, kanamycin, streptomycin, neomycin, and vancomycin resistant	Spades (t149)	89	1,984,518	253,118	76,265	27,714
L. jensenii	VA04-2AN	Trimethoprim resistant	Spades (t145)	83	1,681,921	145,175	56,675	19,479
L. paracasei	VA02-1AN	Chloramphenicol and vancomycin resistant	Spades (t145)	135	3,044,979	434,877	111,561	25,204
L. reuteri	VA24-5	Vancomycin and trimethoprim resistant	Spades (t145)	94	2,179,946	238,354	128,326	26,629
B. bifidum	VA07-1AN	Streptomycin resistant	Spades (t145)	17	2,162,084	467,960	212,216	76,748

Supplementary Table 3.- General features of the genomes of seven antibiotic resistant LAB strains sequenced in this work.

**Feature/gene(s) coding for**	***L. crispatus* VA50-4AN**	***L. jensenii* VA04-2AN**	***L. paracasei* VA02-1AN**	***L. reuteri* VA24-5**	***L. salivarius* VA40-10**	***B. bifidum* VA07-1AN**
Size (bp)	2,215,244	1,681,921	3,044,979	2,179,946	1,984,518	2,162,084
GC content	36.7	34.3	46.2	38.8	32.8	62.6
No. of coding sequences	2,444	1,594	3,219	2,188	1,984	1,839
No. subsystems in RAST	193	182	238	226	215	200
Resistance to antibiotic and toxic compounds[a]	19	9	17	24	32	23
Penicillin binding proteins	9 (8 class C, 1 class A)	1 (class C)	7 (6 class C, 1 class A)	2 (class C)	1 (class C)	1 (class A)
D-ala-D-ala dipeptide ligase	1 type Y	1 type Y	1 type F	1 type F	1 type F	1 (?)
Antibiotic resistance	n.d.	n.d.	n.d.	n.d.	n.d.	*tet*(W) pseudogene
rRNAs	16S+5S+23S	16S+5S+23S	16S+5S+23S	16S+5S+23S	16S+5S+23S	16S+5S+23S
No. of RNAs	72	61	62	75	71	57
Transposases/integrases/excisionases	38	6	29	16	7	12
Phage-related proteins	38	10	59	22	34	4
Competence-associated proteins	14	16	12	19	12	3
CRISPR-associated loci	2	2	2	2	2	-
Bacteriocins-like substances	n.d.	n.d.	Colicin V	Colicin V	n.d.	n.d.
Toxins	Doc toxin, Protein J	Doc toxin, Toxin HigB	Exfoliative toxin A, Protein J	Toxin HigB, Protein J	Protein J	Protein J

[a]RAST category "Virulence, Disease and Defense", subcategory "Resistance to Antibiotic and Toxic Compounds".

n.d., not detected

Supplementary Table 4.- Analysis of the open reading frames (ORFs) and other features identified around the silent *tet*(W) gene from the streptomycin-resistant *B. bifidum* VA07-1AN.

ORF	5’ end	3’ end[a]	% GC content	No. of aa[b]	Known protein with the highest homology (microorganism)[c]	Identity length/total length (% aa identity)	GenBank accession no.
ORF1	651	1,796	54.1	381	Hypothetical protein (*Bifidobacterium longum*)	378/381 (99%)	WP_013140927.1
ORF2	1,898	2,110	55.9	70	Hypothetical protein (*B. longum* subsp. *longum*)	70/82 (100%)	ADH00692.1
ORF3	2,153	2,629	54.3	158	PcfB family protein (*B. longum*)	158/158 (100%)	WP_013140929.1
ORF4	2,626	4,431	54.7	601	Type IV secretory system conjugative DNA transfer family protein (*B. longum*)	601/601 (100%)	WP_117845844.1
ORF5	4,460	4,687	53.9	75	Hypothetical protein (Terrabacteria group)	75/75 (100%)	WP_006060453.1
ORF6’	5,564	6,433	53.1	289	Truncated tetracycline resistance protein (*B. longum*)	288/289 (99%)	ACD97481.1
ORF6’’	6,406	7,485	53.5	359	Truncated tetracycline resistance ribosomal protection protein Tet(W) (*Actinomyces* spp.)	358/639 (99%)	WP_034502607.1
ORF7	7,536	8,894	51.7	452	MFS transporter (*Bifidobacterium bifidum*)	424/425 (99%)	WP_117405967.1
ORF8	11,384	8,895	60.9	829	Esterase-like activity of phytase family protein (*B. bifidum*)	824/829 (99%)	WP_057081546.1
ORF9	12,829	11,630	63.3	399	Elongation factor Tu (*B. bifidum*)	399/399 (100%)	WP_003815489.1
ORF10	15,132	13,003	62.6	709	Elongation factor G (*B. bifidum*)	709/709 (100%)	WP_003813832.1
ORF11	15,630	15,160	62.8	156	30S ribosomal protein S7 (*Bifidobacterium*)	156/156 (100%)	WP_003813834.1
ORF12	16,007	15,636	62.4	123	30S ribosomal protein S12 (*Bifidobacterium*)	123/123(100%)	WP_003815498.1
ORF13	17,224	18,249	65.0	341	Aminotransferase class I/II-fold pyridoxal phosphate-dependent enzyme (*B. bifidum*)	341/341(100%)	WP_013363697.1
ORF14	19,464	18,274	63.1	396	Glycosyltransferase (*B. bifidum*)	396/396 (100%)	WP_003815502.1
ORF15	23,656	19,802	65.8	1,284	Hypothetical protein (*B. bifidum*)	1,280/1,289 (99%)	WP_074740964.1
ORF16	23,919	25,199	70.8	426	Formate-dependent phosphoribosyl-glycinamide formyltransferase (*B. bifidum*)	426/426 (100%)	WP_022173870.1
ORF17	25,517	26,269	61.9	250	Phosphoribosyl-aminoimidazole-succino-carboxamide synthase (*B. bifidum*)	250/250 (100%)	WP_003813894.1
ORF18	26,477	30,223	65.7	1,248	Phosphoribosyl-formyl-glycinamidine synthase (*B. bifidum*)	1,246/1,248 (99%)	WP_057081549.1
ORF19	30,992	30,330	56.4	220	Hypothetical protein (*B. bifidum*)	218/220 (99%)	WP_061085919.1
ORF20	31,731	31,093	50.1	212	DUF4230 domain-containing protein (*B. bifidum*)	212/212 (100%)	WP_003821830.1
ORF21	32,109	32,930	61.3	273	Hypothetical protein BBNG_01261 (*B. bifidum*)	273/273 (100%)	EFR50713.1
ORF22	32,990	33,202	58.7	70	LysR family transcriptional regulator (*B. bifidum*)	70/70 (100%)	WP_013363704.1
ORF23	33,883	33,506	65.1	125	VOC family protein (*B. bifidum*)	125/125 (100%)	WP_013363705.1
ORF24	34,194	33,976	63.9	72	Hypothetical protein (*B. bifidum*)	72/72 (100%)	WP_047284518.1
ORF25	35,828	34,239	62.5	529	Type VI secretion protein ImpB (*B. bifidum*)	526/529 (99%)	WP_065434215.1
ORF26	36,010	37,518	63.9	502	Amidophosphoribosyl-transferase (*B. bifidum*)	501/502 (99%)	WP_003813913.1
ORF27	37,591	38,628	65.0	345	Phosphoribosyl-formyl-glycinamidine cyclo-ligase (*B. bifidum*)	345/345 (100%)	WP_003813914.1
ORF28	38,757	40,022	64.6	421	Phosphoribosylamine-glycine ligase (*B. bifidum*)	421/421 (100%)	WP_014760478.1

[a]Including start and stop codons.
[b]aa, amino acids.
[c]Color code of the different open reading frames (ORFs): Purple, antibiotic resistance genes; yellow, integrase-, mobilization- and conjugation-associated genes; pale blue, genes encoding transcription regulators; white, genes involved in other processes.

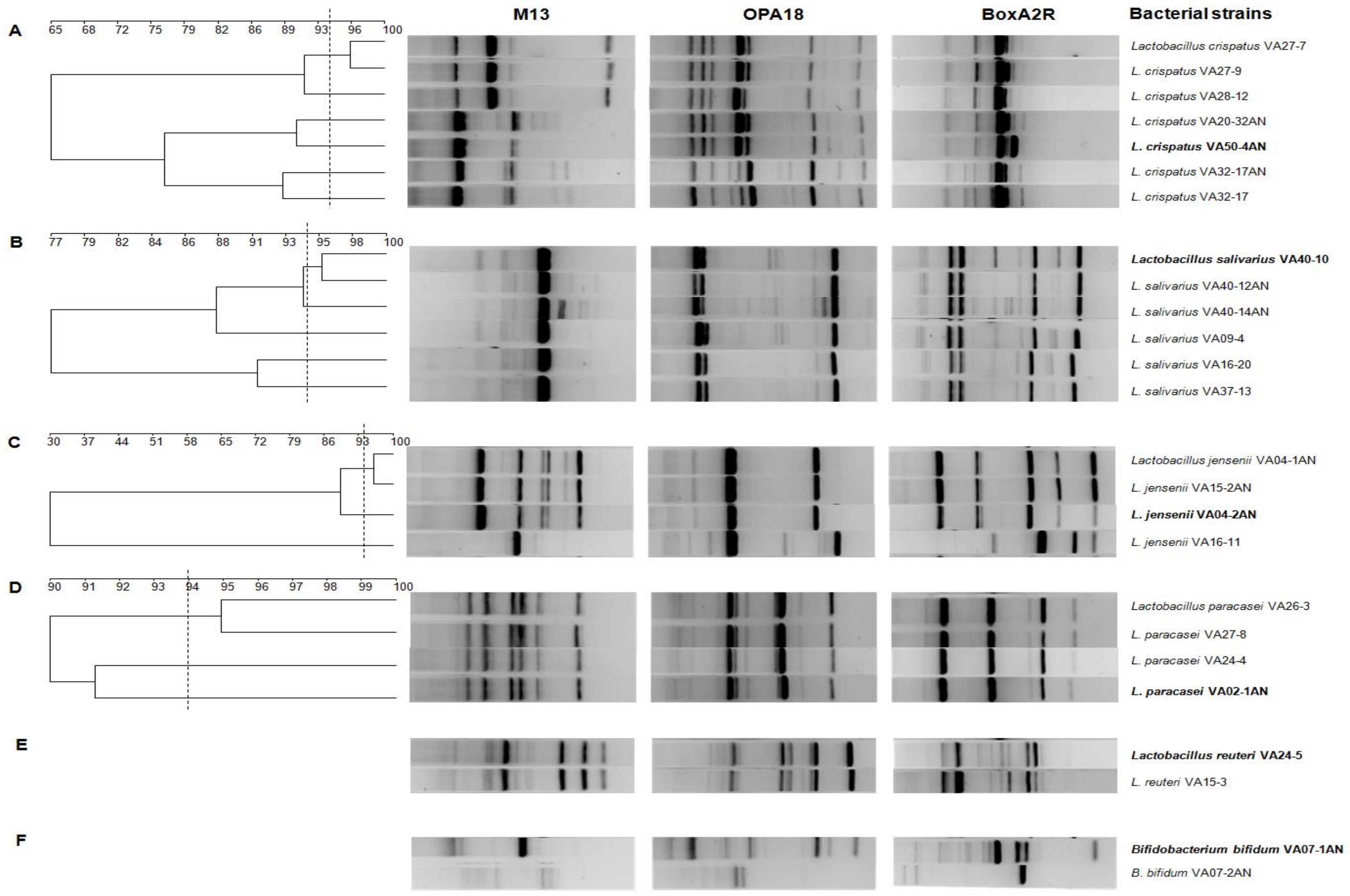

Supplementary Figure 1.- Different rep-PCR and RAPD typing profiles of the LAB strains, obtained with primers M13, OPA18 and BoxA2R, and dendrogram of similarity of the combined typing profiles expressed by the Simple Matching (SM) coefficient of the different strains of this study. Key of the panels: A, strains of *Lactobacillus crispatus;* B, strains of *Lactobacillus salivarius*; C, strains of *Lactobacillus jensenii;* D, strains of *Lactobacillus paracasei;* E, strains of *Lactobacillus reuteri*; and F, strains of *Bifidobacterium bifidum*. Clustering of the profiles was performed by the unweighted pair group method using arithmetic averages (UPGMA). The vertical dotted line indicates 94% correlation (minimum level of reproducibility) as discriminatory at the strain level. The strains shown in bold were selected for subsequent genome sequencing.
